# Use of an innovative colour-based personality-profiling (PP) tool to guide culture-change strategies among different healthcare worker (HCW) groups

**DOI:** 10.1186/2047-2994-4-S1-I9

**Published:** 2015-06-16

**Authors:** L Grayson, N Macesic, C Xuereb

**Affiliations:** 1 Hand Hygiene Australia; 2 Medicine, University of Melbourne, Melbourne, Australia; 3 Infectious Diseases, Austin Health, Heidelberg, Australia; 4 XAX Pty. Ltd., Melbourne, Australia

## Introduction

Most current infection control (IC) culture-change programs are standardised and do not take into account possible differences between HCWs.

## Objectives

1. Identify PP features among various HCW categories to inform the development of a personality-based educational culture-change “blueprint” to improve uptake of IC initiatives.

2. Compare the accuracy of PPs derived from the Human Resources (HR) database at 5 Australian hospitals (HR-Ps) with those derived from direct participant surveys (PS-Ps) from these sites.

3. Use findings to develop targeted marketing strategies for each HCW group.

## Methods

We used an innovative colour-based PP tool (ColourGrid; framework based on Hofstede’s Cultural Dimensions Theory) to identify PPs using: Basic HR data (gender, age, home postcode and suburb, employment status, HCW category) and ColourGrid surveys completed by HCWs at the 5 sites. HR-Ps and PS-Ps were compared for 3 HCW categories – Doctors (D), Nurses/Allied (N-A) and Support staff (SS). Among Ds, PS-Ps were compared for senior hospital clinicians (full-time [SMO] vs part-time [VMO]) and junior staff (interns/fellows [HMO]).

## Results

HR data was obtained for 34 243 HCWs, with 1045 completing a ColourGrid survey. HR-Ps suggested that HCWs are substantially different to the general Australian population, being more affluent; established; well informed; likely to adopt new technology and new experiences; often cynical about advertising messages; challenging to others who do not share their interests or concerns; want to make a difference and leave a heritage of success. HR-Ps and PS-Ps were highly concordant for all 3 HCW categories (D, N-A, SS) – with both suggesting a need for messaging differences. Overall, Ds exhibited more individualism, lower power distance and less uncertainty avoidance, but PS-Ps were different for SMO *vs* VMO *vs* HMO suggesting targeted messaging strategies are critical.

## Conclusion

PP identified major differences among D, N-A and SS; and a need for targeted marketing strategies. Among Ds, subtle but important, differences also exist that need consideration if culture-change initiatives are to be successful.

## Disclosure of interest

None declared.

